# Endoscopic removal using peroral cholangioscopy for biliary migration of vascular coil placed to treat biliary tract bleeding caused by ruptured right hepatic artery aneurysm

**DOI:** 10.1055/a-2864-1385

**Published:** 2026-05-13

**Authors:** Yuichi Hirata, Chikara Akahide, Mariko Hatada, Shunsuke Nakamura, Shinsuke Akiyama, Yoshihide Ueda, Yoshihiro Okabe

**Affiliations:** 1 Department of Gastroenterology 469536 Kakogawa Central City HospitalKakogawaJapan

Hepatic artery aneurysms (HAAs), although rare in patients with infective endocarditis (IE), are associated with life-threatening morbidity and often require endovascular embolization 1 . Biliary coil migration is an infrequent complication reported in the literature and is presumed to occur via a small arterial-biliary fistula 2 . Herein, we report a case of endoscopic removal using peroral cholangioscopy (POCS) for biliary migration of a vascular coil placed to treat biliary tract bleeding caused by rupture of the right HAA.

A 79-year-old man was admitted with abdominal pain. Two years earlier, he had been diagnosed with biliary tract bleeding secondary to the ruptured right HAA complicated by IE and underwent hemostasis through transcatheter coil embolization ( Fig. 1 ). Contrast-enhanced computed tomography demonstrated multiple liver abscesses and bile duct dilation, with an intravascular coil that had migrated into the bile duct and caused obstruction ( Fig. 2 and Fig. 3 ). Magnetic resonance cholangiopancreatography and endoscopic ultrasound confirmed biliary coil migration, with partial adherence of the coil to the bile duct wall ( Fig. 4 ). After prioritizing treatment of cholangitis and liver abscesses, endoscopic retrograde cholangiopancreatography was performed for coil removal. POCS (eyeMAX, Micro-Tech, Nanjing, China) was used to assess feasibility by directly visualizing the relationship between the bile duct wall and the coil. Although a portion of the coil was invaginated into the bile duct wall, most of it remained within the lumen, indicating that safe removal was feasible. A stone complex had formed around the distal end of the coil mass; fragmentation using electrohydraulic lithotripsy exposed the coil. The coil was grasped with biopsy forceps under POCS and successfully removed, followed by extraction of residual stone fragments. Final POCS confirmed the absence of residual coils and stones ( Fig. 5 ; Video 1 ).

**Fig. 1:**
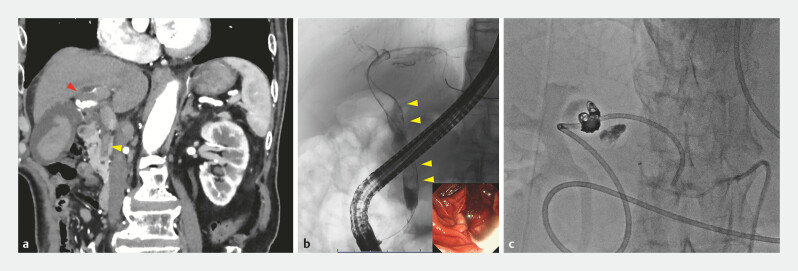
Computed tomography, fluoroscopy and angiography. **a** Two years earlier, he had been diagnosed with biliary tract bleeding secondary to the ruptured right HAA complicated by IE (red arrowhead). Hemobilia was found in the common bile duct (CBD; yellow arrowhead). **b** The CBD was filled with hematoma. **c** He underwent hemostasis through transcatheter coil embolization. HAA, hepatic artery aneurysm; IE, infective endocarditis.

**Fig. 2:**
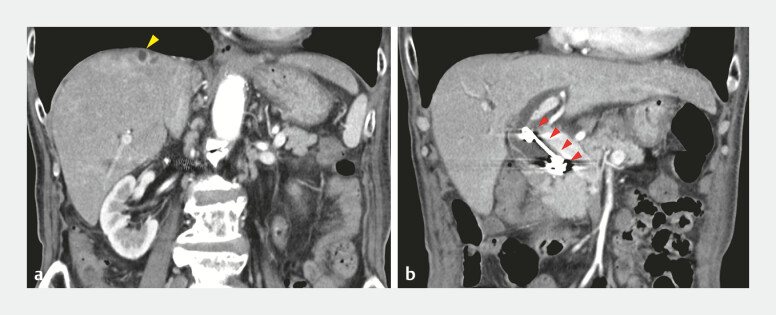
Computed tomography. Contrast-enhanced computed tomography demonstrated multiple liver abscesses (yellow arrowhead) and bile duct dilation, with an intravascular coil that had migrated into the bile duct and caused obstruction (red arrowhead).

**Fig. 3:**
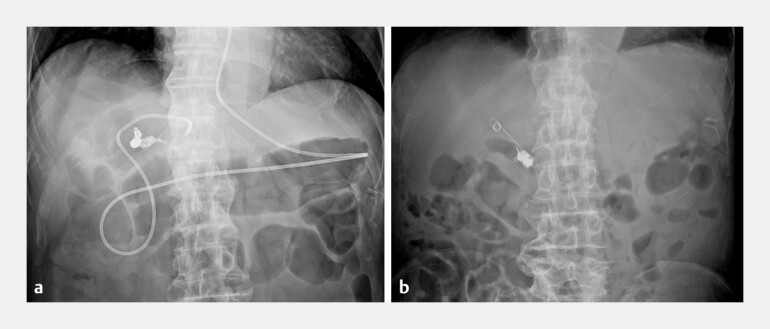
X-ray images. **a** Two years earlier. **b** This time.

**Fig. 4:**
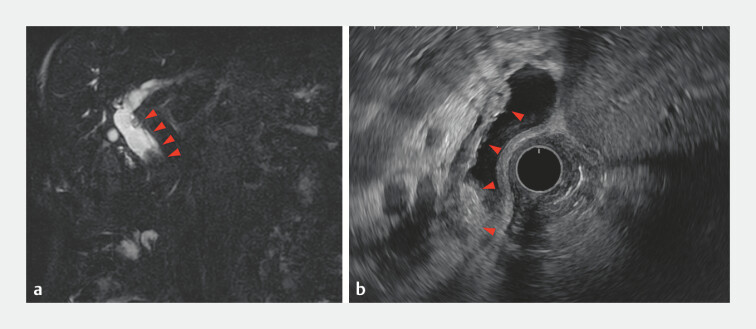
Magnetic resonance cholangiopancreatography (MRCP) and endoscopic ultrasound (EUS). These examinations confirmed biliary coil migration, with partial adherence of the coil to the bile duct wall. **a** MRCP and **b** EUS.

**Fig. 5:**
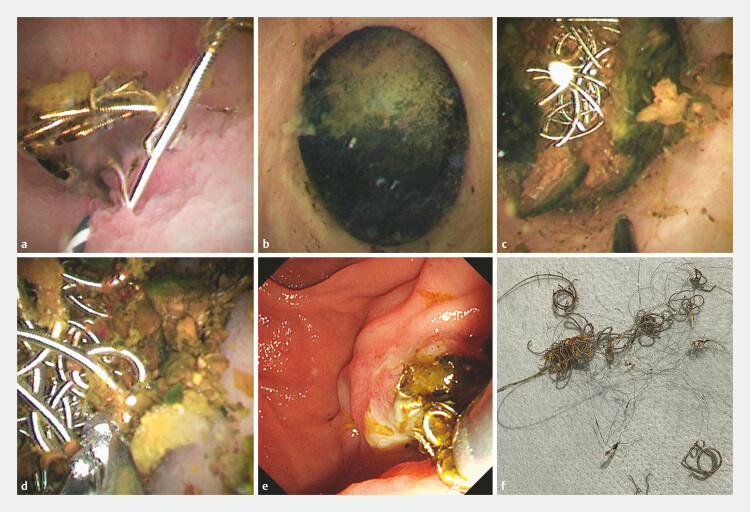
Peroral cholangioscopic (POCS) images and removed coil. **a** A portion of the coil was invaginated into the bile duct wall. **b** A stone complex had formed around the distal end of the coil mass. **c** and **d** Fragmentation using electrohydraulic lithotripsy exposed the coil. **e** The coil was grasped with biopsy forceps under POCS and successfully removed. **f** Removed coil.

Endoscopic removal using peroral cholangioscopy for biliary migration of a vascular coil placed to treat biliary tract bleeding caused by rupture of the right hepatic artery aneurysm.Video 1

Although coil removal using percutaneous transhepatic cholangioscopy has been reported 3 , this represents the first case utilizing POCS.

Endoscopy_UCTN_Code_CPL_1AM_2AZ

## Notes

Correction **Correction: Endoscopic removal using peroral cholangioscopy for biliary migration of vascular coil placed to treat biliary tract bleeding caused by ruptured right hepatic artery aneurysm** Hirata Yuichi, Akahide Chikara, Hatada Mariko et al. Endoscopic removal using peroral cholangioscopy for biliary migration of vascular coil placed to treat biliary tract bleeding caused by ruptured right hepatic artery aneurysm. Endoscopy 2026; 58: E606–E608, doi:10.1055/a-2864-1385 In the above-mentioned article the title has been corrected. The correct title is: Endoscopic removal using peroral cholangioscopy for biliary migration of vascular coil placed to treat biliary tract bleeding caused by ruptured right hepatic artery aneurysm. This was corrected in the online version on May 19, 2026.
