# Correction: Endoscopic removal using peroral cholangioscopy for biliary migration of vascular coil placed to treat biliary tract bleeding caused by ruptured right hepatic artery aneurysm

**DOI:** 10.1055/a-2877-6222

**Published:** 2026-05-19

**Authors:** Yuichi Hirata, Chikara Akahide, Mariko Hatada, Shunsuke Nakamura, Shinsuke Akiyama, Yoshihide Ueda, Yoshihiro Okabe

**Affiliations:** 1 Department of Gastroenterology 469536 Kakogawa Central City HospitalKakogawaJapan



In the above-mentioned article the title has been corrected. The correct title is: Endoscopic removal using peroral cholangioscopy for biliary migration of vascular coil placed to treat biliary tract bleeding caused by ruptured right hepatic artery aneurysm. This was corrected in the online version on May 19, 2026.

